# Posttreatment Changes in Cytokines Induced by *Schistosoma mansoni* Egg and Worm Antigens: Dissociation of Immunity- and Morbidity-Associated Type 2 Responses

**DOI:** 10.1093/infdis/jit826

**Published:** 2013-12-19

**Authors:** Shona Wilson, Frances M. Jones, Lee-Carol Kenty, Joseph K. Mwatha, Gachuhi Kimani, H. Curtis Kariuki, David W. Dunne

**Affiliations:** 1 Department of Pathology, University of Cambridge, United Kingdom; 2 Kenya Medical Research Institute; 3 Division of Vector Borne Diseases, Ministry of Health, Nairobi, Kenya

**Keywords:** *Schistosoma mansoni*, human, cytokine, praziquantel, immunity, immunopathology

## Abstract

***Background.*** Human type 2 cytokine responsiveness to schistosome antigens increases after treatment; due either to removal of the immunosuppressive effects of active infection or immunological boosting by antigens released from dying parasites. We determined the responsiveness to *Schistosoma mansoni* over a 2-year period, when reinfection was restricted by interrupting transmission.

***Methods.*** The proinflammatory and type 2 responses of Kenyan schoolchildren were measured before, and 1 year and 2 years posttreatment in whole blood cultures stimulated with soluble egg antigen (SEA) or soluble worm antigen (SWA). The site of *S. mansoni* transmission was molluscicided throughout.

***Results.*** Pretreatment proinflammatory responses to SEA were high but reduced 1 and 2 years posttreatment, whereas type 2 responses were low pretreatment and increased 1 and 2 years posttreatment. Type 2 responses to SWA were high pretreatment and increased at 1 year, with no further increases at 2 years posttreatment. Children infected at follow-up had lower SEA, but not SWA, posttreatment type 2 responsiveness. Increases at 1 year in type 2 SWA, but not SEA, responsiveness correlated with pretreatment egg counts.

***Conclusions.*** Removal of immunosuppressive effects of active infection increases SEA type 2 responsiveness; long-term SWA type 2 responsiveness is due to treatment-induced immunological boosting. Dissociation of type 2 responses potentially protects against severe egg-associated immunopathology during infection, while allowing worm-antigen derived immunity to develop.

Schistosomiasis causes extensive loss of quality of life, particularly in sub-Saharan Africa [1], where *Schistosoma mansoni* is 1 of the 2 most prevalent schistosome infections. Morbidity associated with *S. mansoni* is caused by the immune response to eggs that are swept into the liver, becoming trapped in the tissues. In murine models of *S. mansoni* infection, a strong type 2 response is initially mounted against the eggs but is down-modulated as infection becomes chronic [2]. In chronically infected humans, low proliferative and type 2 cytokine responsiveness to soluble egg antigen (SEA) indicate that T cells are hyporesponsive to eggs [3, 4]. The severest manifestation of infection, periportal fibrosis, is associated with failure to down-regulate this type 2 responsiveness [5–7]. In contrast, cells from children chronically infected with *S. mansoni* who present with hepatosplenomegaly in the absence of periportal fibrosis produce high levels of the type 1 proinflammatory cytokine tumor necrosis factor alpha (TNF-α) in response to SEA stimulation [8, 9].

In chronically infected individuals, high type 2 cytokine levels have been observed in response to soluble worm antigen (SWA) [10]. The levels of the type 2 cytokine interleukin (IL) 5, in pretreatment SWA-stimulated cultures [11] and in plasma after treatment-induced in vivo exposure to antigen from dying worms [12] have been linked to protective IgE responses, protective responses that require progressive accumulation of worm/cercariae cross-reactivity [13]. Treatment of schistosomiasis increases T-cell proliferation after SWA stimulation, suggesting that T cells may also be SWA hyporesponsive [3, 14]. However, it has also been proposed that exposure to previously cryptic antigens in the worm, upon treatment with praziquantel, boosts SWA-specific type 2 cytokine responses [15, 16].

Because the host needs to down-modulate the Th2 response to eggs to prevent immunopathology, but maintain this response to adult worm antigens during development of immunity to further infection, it has been proposed that regulation of the type 2 response to differing life stages of the parasite is dissociated [17]. However, as reinfection after treatment occurs rapidly in endemic areas, it is difficult to study the influence of current infection or long-term infection-free responses in treated cohorts. We conducted a 2-year longitudinal study of responses to SEA and SWA in which transmission was kept to a minimum by treating the whole community and molluscide treating the sole habitat of the intermediate snail host within the study area, thus allowing us to study responses annually, in the absence of high levels of reinfection and antigen exposure upon retreatment.

## MATERIALS AND METHODS

### Study Area, Population, and Design

**Figure 1.**
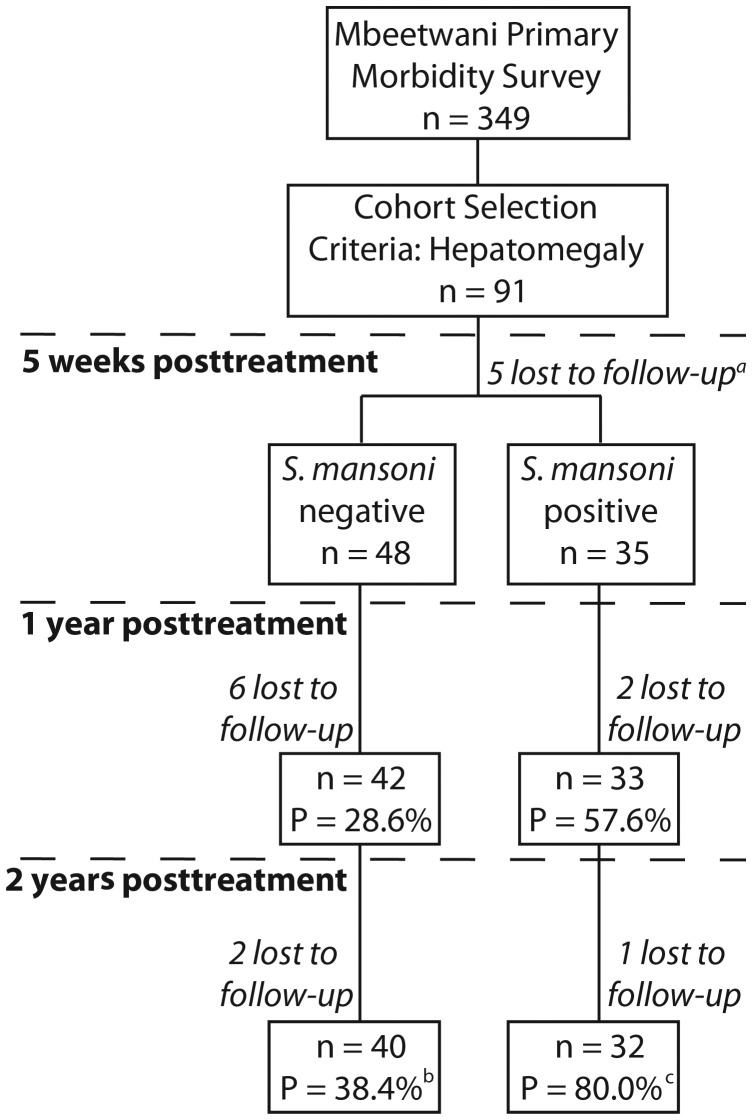
Cohort recruitment and follow-up. Shown is the treatment efficacy at 5 weeks, and the success of follow-up and prevalence (P) of *Schistosoma mansoni* at 1 year and 2 years posttreatment. ^a^Three individuals successfully followed up at 1 and 2 years posttreatment did not provide stool samples at 5 weeks. ^b^Prevalence based on 39 individuals and ^c^30 individuals, due to missing parasitological data among those followed up immunologically at 2 years posttreatment.

Ninety-one Akamba schoolchildren, 43 boys and 48 girls, aged 7–18 years (mean, 11.95 years), attending Mbeetwani Primary School, Makueni District, Kenya, participated in the study (Figure 1). Children were selected on the basis of presentation with hepatomegaly at baseline, to assess regression of morbidity and corresponding immunological measurements. None had ultrasound-detectable periportal fibrosis [18]. Three stool samples were collected from each participant and 2 Kato-Katz slides were prepared from each stool for *S. mansoni* egg counts. All participants were treated with 40 mg/kg praziquantel after samples were collected. Three stools were collected at 5 weeks posttreatment from 83 participants to assess efficacy of treatment.

Seventy-eight children, 38 girls and 40 boys (mean age at baseline, 11.78 years) were followed up 1 year posttreatment, of whom 75 (36 girls and 39 boys; mean age at baseline, 11.71 years) were followed up 2 years posttreatment. At each follow-up, participants provided 3 stools for *S. mansoni* egg counts and received a further praziquantel treatment. The rest of the community was treated immediately after baseline activities. For the duration of the study, the Kambu River, the only source of *S. mansoni* transmission in Mbeetwani, was regularly treated with the molluscide Bayluscide to limit reinfection of the cohort. Detailed descriptions of the study design, including molluscide application, and morbidity resolution are given elsewhere [18–20].

The study received ethical clearance from the Kenya Medical Research Institute ethical review committee. The purpose of the study was carefully explained to members of the community and consent was given by the parents/guardians of the participants.

### Whole Blood Cultures

Details of SEA and SWA preparation is described elsewhere [10]. SEA and SWA were filtered through sterile 0.22-µm pore-size filters and endotoxin assayed using the *Limulus* amebocyte lysate kit (QCL-100, BioWhittaker, Inc). Purified endotoxin at the concentration found in 10 µg of SEA or SWA preparations (<0.3 ng/mL) does not stimulate detectable production of any of the cytokines measured. The same preparation batches of antigen were used at all time points.

Prior to treatment, venous blood was collected into heparin at 10 U/mL. One milliliter of whole blood cultures (diluted 1:6 in RPMI 1640) stimulated with SEA, SWA, phytohemagglutin (PHA), and unstimulated control cultures, was set up in biological duplicate. Each culture contained 10 μg/mL antigen, with 5 U/mL penicillin, 50 μg/mL streptomycin, and 2 mM l-glutamine. Cultures were incubated at 37°C for 96 hours. Eight hundred microliters of supernatant were harvested. Supernatants were treated with 0.3% tributyl phosphate/1% Tween80 (both Sigma) to inactivate encapsulated viruses prior to being assayed.

### Cytokine and Chemokine Assays

Eotaxin, interferon gamma (IFN-γ), TNF-α, IL-4, IL-5, IL-9, IL-10, IL-12p40, IL-13, IL-21, and IL-33 were measured using multiplex Luminex bead technology. In brief, COOH beads were coupled to capture antibodies (Abs), incubated overnight at 4°C with 25 μL of supernatant, diluted 1:2, and detected with matched detecting Abs. The assay sensitivity was 0.02 pg/mL for all cytokines/chemokine, except for IL-12p40 for which it was 0.2 pg/mL and IL-13 for which it was 0.06 pg/mL. IL-1β, IL-6, and CCL5 were measured by enzyme-linked immunosorbent assay using matched Ab pairs, with an overnight incubation with supernatants at 4°C. Supernatants were diluted 1:10 for IL-1β, 1:20 for CCL5 and 1:100 for IL-6. Assay sensitivity cutoffs were assigned to individuals when levels of the cytokine/chemokine were undetectable. Abs for IFN-γ, TNF-α, IL-1β, IL-4, IL-5, IL-6, IL-9, IL-10, IL-12p40, IL-13, and IL-21 were purchased from BD Pharmingen. Abs for IL-33, eotaxin, and CCL5 were purchased from R&D Systems.

### Statistical Analysis

Comparisons of pretreatment responses to differing antigens and longitudinal responses to the same antigen were analyzed by nonparametric paired Wilcoxon tests. Pretreatment, 5 comparisons (media vs SEA, media vs SWA, SEA vs SWA, SEA vs PHA, and SWA vs PHA) were made for each of 11 cytokines/chemokines. Twenty-three comparisons were made for longitudinal SEA responses: pretreatment vs 1 year posttreatment and 1 year vs 2 years posttreatment for 11 cytokines/chemokines, and an additional comparison of pretreatment vs 2 years posttreatment for IFN-γ levels. For longitudinal comparisons of SWA responses 14 tests were carried out: pretreatment vs 1 year posttreatment and 1 year vs 2 years posttreatment for 7 cytokines. The cohort was divided into age groups: 7–10 years (n = 32), 11–13 years (n = 30), and 14–18 years (n = 29) and responses by age group were analyzed by Kruskal–Wallis tests. Comparison of cytokine responses by infection status at 1 and 2 years posttreatment were analyzed by Mann–Whitney *U* tests. Correlations were calculated between changes in cytokine/chemokine responses (1 year posttreatment – pretreatment) and pretreatment infection intensity using Spearman rank correlation. Individual test statistics for significant differences (Supplementary Tables 1–3) are not presented in the text due to the number of tests carried out. Simes-modified Bonferroni correction for multiple testing was applied, and all differences described reached the required level of significance unless otherwise specified.

## RESULTS

### Parasitology

The pretreatment prevalence of *S. mansoni* was 79.1% and the median infection intensity was 85 eggs/g feces (epg), with an interquartile range (IQR) of 5.0–208.3 epg. Prevalence was reduced to 42.2% at 5 weeks posttreatment, and the infection intensity median was zero (IQR, 0–5.83 epg). Regular mollusciding of the Kambu River maintained similar infection levels 1 year posttreatment, when the prevalence of infection was 41.0% and the median infection intensity remained zero (IQR, 0–6.67 epg). At 2 years posttreatment, the prevalence had increased slightly to 56.9%, but the infection intensities remained low at a median of 3.33 epg (IQR, 0–10.0 epg).

### Pretreatment Responses

IL-21 and IL-33 were not detectable for the majority of individuals, with 76 (83.5%) and 78 (85.7%) individuals having undetectable levels of IL-21 release to SEA and SWA, and 66 (72.5%) and 75 (82.4%) individuals having undetectable IL-33 release to SEA and SWA, respectively. Thirty-six (39.6%) individuals had no detectable eotaxin in SEA-stimulated cultures and 34 (37.4%) had no detectable eotaxin in SWA-stimulated cultures. Overall eotaxin release in SEA- and SWA-stimulated cultures was not significantly higher than in the unstimulated cultures (*U* = 1443.5, *P* = .3444 and *U* = 1545.5, *P* = .077, respectively).

**Figure 2.**
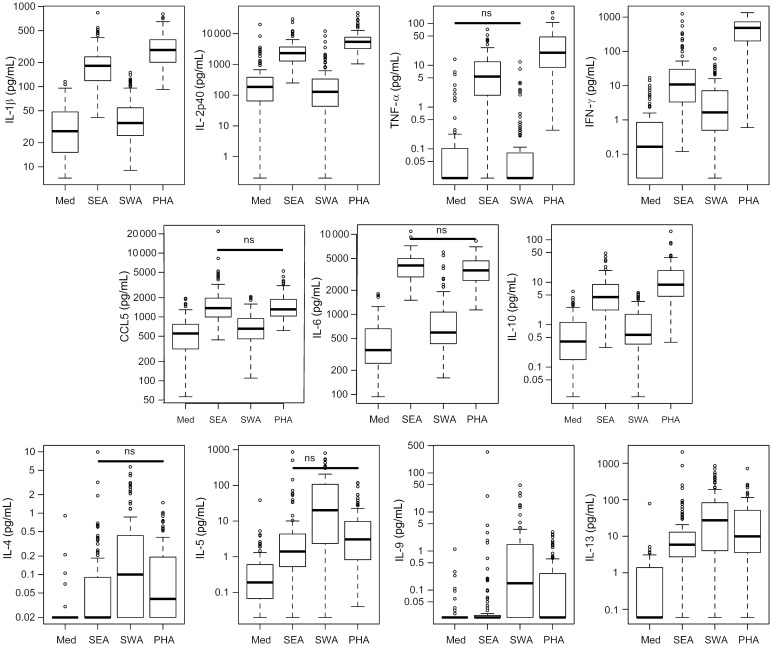
Cytokine production in whole blood cultures stimulated with *Schistosoma mansoni* antigens and the mitogen phytohemagglutin (PHA). The levels of cytokines and the chemokine CCL5 were measured in pretreatment whole blood culture supernatants left unstimulated (Med), or stimulated with soluble egg antigen (SEA), soluble worm antigen (SWA), or with the mitogen PHA. The boxes show the medians and interquartile ranges of the cytokine levels, with whiskers representing up to 1.5 times the interquartile range. Circles represent outlying data points. Nonsignificant (ns) comparisons using paired Wilcoxon tests are indicated. All other comparisons remained significant after correction for multiple testing by Simes-modified Bonferroni method. Abbreviations: IFN-γ, interferon gamma; IL, interleukin; TNF-α, tumor necrosis factor alpha.

The levels of the other cytokines, and chemokine CCL5, measured in response to SEA, SWA, and PHA and in unstimulated cultures are shown in Figure 2. In SWA-stimulated cultures, 54 individuals (59.3%) had no detectable TNF-α. Additionally, TNF-α release was not significantly higher in SWA-stimulated cultures than in unstimulated cultures (*U* = 381, *P* = .16). In contrast, TNF-α was measurable for all but 2 individuals in cultures stimulated with SEA. The release of TNF-α was significantly higher in cultures stimulated with SEA than in the unstimulated and SWA-stimulated cultures. The same pattern was observed for IL-1β, IL-12p40, IL-6, CCL5, and IL-10, with responses in SEA-stimulated cultures being significantly higher than in SWA-stimulated cultures; however, with the exception of IL-12p40, these were all measurable at significantly higher levels in SWA-stimulated cultures than in unstimulated cultures. Levels of IL-12p40 were significantly lower in SWA-stimulated cultures than in unstimulated cultures. PHA-stimulated cultures produced significantly more IL-12p40, TNF-α, and IL-10 than did SEA- and SWA-simulated cultures, but comparable amounts of IL-6 (*U* = 253, *P* = .082) and CCL5 (*U* = 1663, *P* = .122) were measurable in SEA- and PHA-stimulated cultures. In SEA- and SWA-stimulated cultures, IFN-γ levels were significantly higher than in unstimulated cultures. There was significantly more IFN-γ produced in SEA-stimulated cultures than SWA-stimulated cultures. More IFN-γ was produced in response to PHA than to SEA.

The number of individuals with measurable IL-4 and IL-9 responses was low, particularly for SEA, for which 51 individuals (56%) and 68 individuals (74.7%) had undetectable IL-4 and IL-9. For SWA, 32 (35.3%) and 34 (37.4%) individuals had undetectable IL-4 and IL-9. However, the IL-4 and IL-9 responses were significantly higher for both SEA- and SWA-stimulated cultures when compared with unstimulated cultures. IL-5 and IL-13 responses were also higher in SEA- and SWA-stimulated cultures when compared with unstimulated cultures. For IL-4, IL-5, IL-9, and IL-13, the responses measured in SWA-stimulated cultures were significantly higher than those measured in SEA- and PHA-stimulated cultures.

There was no significant difference in pretreatment cytokine responses to SEA (*P* > .127) or SWA (*P* > .060) for the different age groups, with the exception of SWA-IL-4 (χ^2^ = 9.022, *P* = .011), which was no longer significant after correction for multiple testing (*P* < .005 required).

### Posttreatment SEA Responses

**Figure 3.**
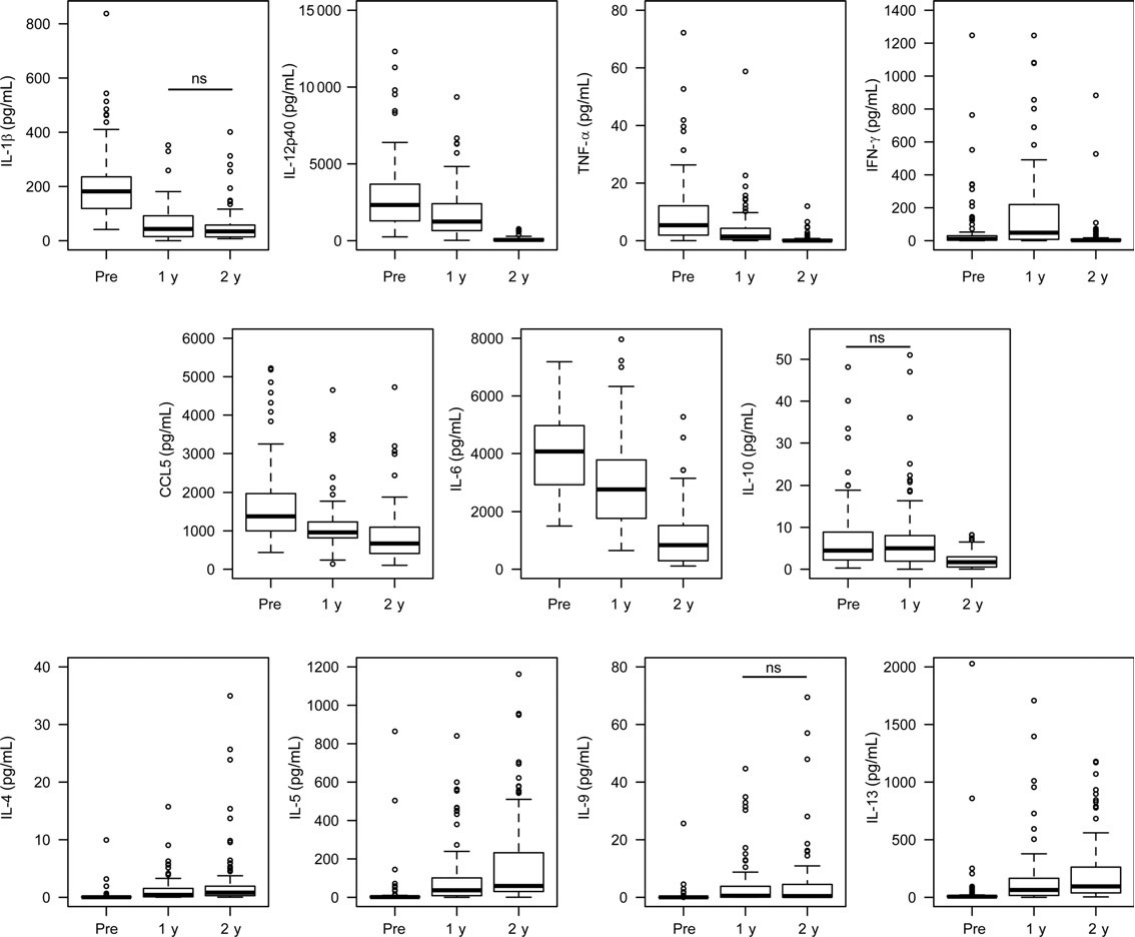
Longitudinal cytokine production in whole blood cultures stimulated with *Schistosoma mansoni* soluble egg antigen (SEA). Shown are the levels of cytokines and the chemokine CCL5 measured in whole blood cultures stimulated with SEA pretreatment, 1 year posttreatment, and 2 years posttreatment. Nonsignificant (ns) comparisons using paired Wilcoxon tests are indicated. All other longitudinal comparisons remained significant after correction for multiple testing by Simes-modified Bonferroni method. Abbreviations: IFN-γ, interferon gamma; IL, interleukin; TNF-α, tumor necrosis factor alpha.

IL-1β, IL-12p40, TNF-α, CCL5, and IL-6 responses all significantly decreased in SEA-stimulated cultures 1 year posttreatment in comparison with pretreatment cultures (Figure 3). These responses in 2 years posttreatment SEA-stimulated cultures were significantly lower again compared with 1 year posttreatment, except for IL-1β (*U* = 1187, *P* = .281). In contrast, IFN-γ responses in SEA-stimulated cultures were significantly higher 1 year posttreatment in comparison with pretreatment, but at 2 years posttreatment were lower than both pretreatment and 1 year posttreatment. IL-10 responses in SEA-stimulated cultures were not significantly lower 1 year posttreatment compared to pretreatment (*U* = 1386, *P* = .443), but were significantly lower 2 years posttreatment than at 1 year posttreatment.

There were significant increases in all the type 2 cytokine responses, IL-4, IL-5, IL-9, and IL-13, in SEA-stimulated cultures between pretreatment and 1 year posttreatment (Figure 3). The IL-4, IL-5, and IL-13 responses continued to increase between 1 year and 2 years posttreatment. IL-9 responses were not significantly different 2 years posttreatment in comparison with 1 year posttreatment (*U* = 951, *P* = .427).

There were no significant correlations between the decrease in proinflammatory responses and the increase in type 2 responses in SEA-stimulated cultures pretreatment to 1 year posttreatment (data not shown).

### Posttreatment SWA Responses

At 1 year posttreatment, in addition to TNF-α and IL-12p40, IL-1β (*U* = 1638, *P* = .261) and CCL5 (*U* = 1397, *P* = .476) were not significantly higher in SWA-stimulated cultures than in unstimulated control cultures, so longitudinal trends in these proinflammatory cytokines/chemokine were not analyzed.

IL-6 in SWA-stimulated cultures at 1 year posttreatment was significantly lower than in pretreatment cultures, but 2 years posttreatment was not significantly less than in 1 year posttreatment cultures (*U* = 1745, *P* = .054). Levels of IFN-γ did not change pretreatment to 1 year posttreatment in response to SWA (*U* = 4125, *P* = .070), or 1 year posttreatment to 2 years posttreatment (*U* = 3141, *P* = .745); nor were any significant differences in IL-10 responses to SWA observed: pretreatment vs 1 year posttreatment (*U* = 3436, *P* = .723) and 1 year vs 2 years posttreatment (*U* = 991, *P* = .048, significance of *P* = .025 required due to multiple testing).

**Figure 4.**
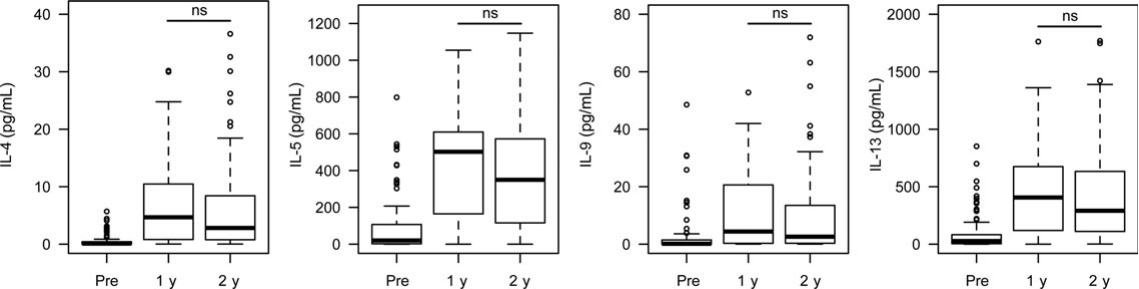
Longitudinal type 2 cytokine production in whole blood cultures stimulated with *Schistosoma mansoni* soluble worm antigen (SWA). Shown are the levels of type 2 cytokines measured in whole blood cultures stimulated with SWA pretreatment, 1 year posttreatment, and 2 years posttreatment. Nonsignificant (ns) comparisons using paired Wilcoxon tests are indicated. All other longitudinal comparisons remained significant after correction for multiple testing by Simes-modified Bonferroni method. Abbreviation: IL, interleukin.

The type 2 cytokines IL-4, IL-5, IL-9 and IL-13 were all significantly higher in SWA-stimulated cultures 1 year posttreatment compared to pretreatment cultures (Figure 4). There was no significant difference in these responses at 2 years posttreatment in comparison with 1 year posttreatment: IL-4 (*U* = 1731, *P* = .065), IL-5 (*U* = 1769, *P* = .070), IL-9 (*U* = 1427, *P* = .040, significance of .021 required due to multiple testing), and IL-13 (*U* = 1662, *P* = .212).

### Longitudinal Type 2 Cytokine Responses and Parasitological Factors

**Figure 5.**
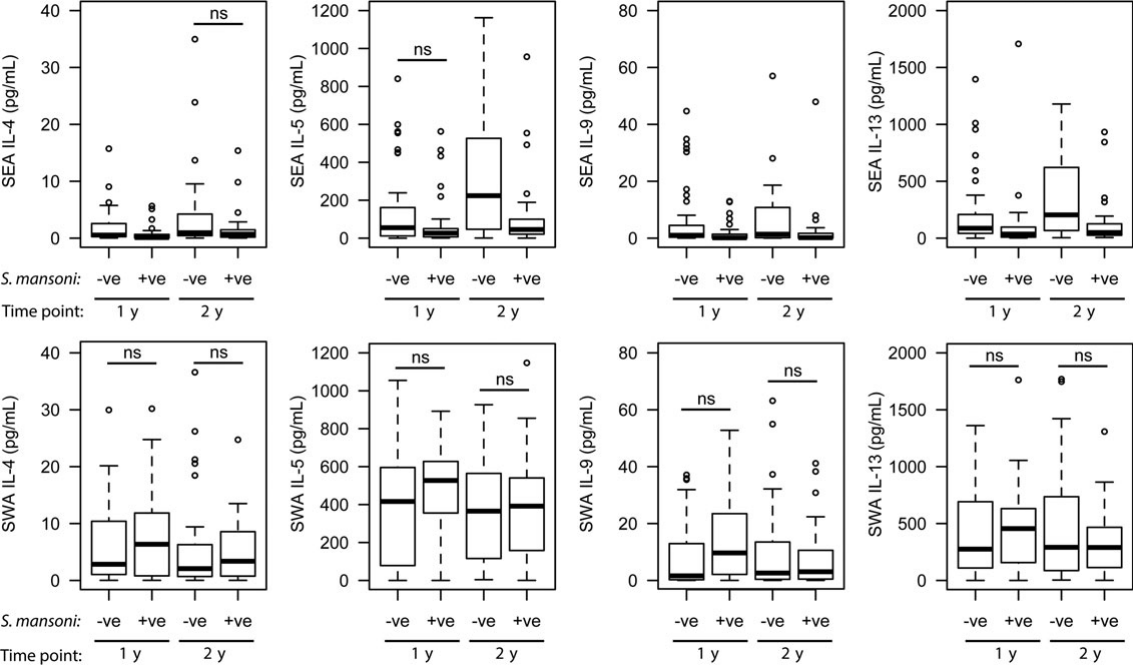
Posttreatment type 2 cytokine production in whole blood cultures stimulated with schistosome antigens in children positive and negative for *Schistosoma mansoni* infection. Shown are the levels of type 2 cytokines measured in whole blood cultures stimulated with either soluble egg antigen (SEA) or soluble worm antigen (SWA), 1 year and 2 years posttreatment for schistosomiasis, for children who did (1 year posttreatment [n = 32], 2 years posttreatment [n = 42]) and did not have detectable infections (1 year posttreatment [n = 46] and 2 years posttreament [n = 31]) at those time points. Nonsignificant (ns) comparisons using Mann–Whitney tests are indicated. All other comparisons remained significant after correction for multiple testing by Simes-modified Bonferroni method. Abbreviations: –ve, negative; +ve, positive; IL, interleukin.

To examine whether the same mechanism was contributing to the increase in type 2 responses to SEA and SWA posttreatment, they were analyzed in relation to the parasitological data. Figure 5 shows the IL-4, IL-5, IL-9, and IL-13 responses measured in SEA- and SWA-stimulated cultures at 1 year and 2 years posttreatment among children who did and did not have detectable *S. mansoni* infection at that time point. At both 1 year posttreatment and 2 years posttreatment, the type 2 cytokine responses to SEA were higher among children who had no detectable *S. mansoni* infection. The exceptions to this were IL-5 at 1 year posttreatment (*U* = 855, *P* = .052) and IL-4 at 2 years posttreatment (*U* = 817, *P* = .100). In contrast, there was no significant difference in the IL-4, IL-5, IL-9, and IL-13 responses to SWA between children who did and did not have detectable *S. mansoni* infections at either 1 year (*P* > .125) or 2 years (*P* > .472) posttreatment.

The IL-4, IL-5, IL-9, and IL-13 pretreatment responses were subtracted from 1 year posttreatment responses, and correlations between the changes and pretreatment infection intensity, a proxy of the amount of worm-derived antigen exposure upon treatment, were calculated (Table 1). None of the increases in IL-4, IL-5, IL-9, and IL-13 in response to SEA were correlated with pretreatment infection intensity. However, there were significant positive correlations between the increase in all 4 of the type 2 cytokines in response to SWA and pretreatment infection intensities.

**Table 1.** Correlations Between Pretreatment Infection Intensity and Pretreatment to 1-Year Posttreatment Increases in Type 2 Cytokine Release

	SEA		SWA	
Cytokine	ρ	*P* Value	ρ	*P* Value
IL-4	0.030	.792	0.326	.004
IL-5	−0.056	.626	0.361	.001
IL-9	−0.190	.100	0.254	.025
IL-13	−0.037	.747	0.258	.023

Increases in cytokine release were calculated by subtracting the levels measured in pretreatment cultures from the levels measured in the 1-year posttreatment cultures and were correlated with pretreatment egg counts using Spearman rank correlation. All significant correlations remained after Simes-modified Bonferroni correction for multiple testing (8 tests).

Abbreviations: IL, interleukin; SEA, soluble egg antigen; SWA, soluble worm antigen.

\

## DISCUSSION

The current study examined the long-term proinflammatory, type 1, regulatory, and type 2 responses to differing *S. mansoni* life stages of previously chronically exposed schoolchildren for whom reinfection was kept to a minimum by molluscide treatment of the source of transmission. This allowed examination of the role of active infection on maintaining hyporesponsiveness, in the absence of high reinfection. While IL-21, IL-33, and eotaxin were undetectable, the significant patterns that were found for other cytokines/chemokines show dissociation of the type 2 responses to SEA and SWA.

The hyporesponsiveness of T cells in chronic human schistosome infection is a reproducible phenomenon, with treatment recovering cellular proliferation to SEA, suggesting that active infection is required to maintain hyporesponsiveness [3]. In the current study, those children for whom infections were detectable at follow-up, both at 1 year and 2 years posttreatment, had lower type 2 responses to SEA, again indicating that Th2 cell hyporesponsiveness is associated with active exposure to egg antigens. This down-modulation will be essential in minimizing the progression of morbidity to periportal fibrosis, the severest manifestation of infection, as it is associated with long-term exposure to eggs [21] and higher levels of Th2 responsiveness to SEA [7].

Hepatosplenomegaly associated with *S. mansoni* infection, in the absence of ultrasound detectable periportal fibrosis, is associated with TNF-α production and poor regulatory responses [9], suggesting that the active down-regulation of anti-inflammatory Th2 responses may have pathological consequences by allowing proinflammatory responses to expand. In the current study, all participants were part of a cohort selected on the basis of having hepatomegaly, but none had ultrasound-detectable periportal fibrosis [18]. Pretreatment, predominant release of proinflammatory cytokines in response to SEA was observed. With treatment and removal of Th2 hyporesponsiveness, the strength of this proinflammatory response declined, and continued to decline over the course of the 2-year follow-up period.

Although IFN-γ was at higher levels in response to SEA than SWA pretreatment, this cytokine did not follow the same pattern longitudinally as IL-1β, TNF-α, CCL5, and IL-6, with SEA-specific levels increasing 1 year posttreatment, prior to decreasing 2 years posttreatment. The reasons for this difference are not known, but it does indicate that the predominant proinflammatory response observed pretreatment, which is then down-regulated posttreatment, is not associated with an adaptive Th1 response, of which IFN-γ is an archetypal cytokine. We have also previously detected no IL-17 in response to SEA for a similar cohort of Kenyan schoolchildren [9]. It is therefore likely that the proinflammatory cytokines are derived from innate immune cells. Understanding of the role of innate immune cells in schistosomiasis is increasing, with egg-derived glycoproteins interacting with Toll-like receptors (TLRs) and c-type lectins [22, 23]. Although reduced responsiveness of myeloid dendritic cells upon TLR engagement has been proposed to contribute T-cell hyporesponsiveness [24], increased proinflammatory cytokine responses by peripheral blood mononuclear cells stimulated with TLR ligands have been reported for children infected with *S. haematobium* [25]. It should be noted that the predominance of proinflammatory pretreatment responses to SEA observed in the current study may reflect the selection procedure, in which presentation with hepatomegaly was a criterion. Adults without ultrasound-detectable periportal fibrosis have reduced rates of hepatosplenomegaly compared to children [26] and have been shown to release only low levels of TNF-α in response to both SEA and SWA stimulation [10].

T-cell hyporesponsiveness has also been reported for the response against SWA [3, 27], and as some antigens are expressed by both the egg and the adult worms [28], T cells specific for these shared antigens are likely to be hyporesponsive. However, in the current study, high type 2 responses to SWA were observed pretreatment, so Th2 cell hyporesponsiveness is less apparent in the response to SWA than to SEA. This concurs with the higher pretreatment cellular proliferation and IL-5 levels after SWA stimulation compared to SEA stimulation that has previously been reported [29]. As the levels of type 2 cytokines—IL-4, IL-5, IL-9, and IL-13—were higher in the whole blood cultures stimulated with SWA than PHA (a mitogen that works through the interaction between antigen-presenting cells and T cells), other cells present, such as eosinophils, may be releasing these cytokines or up-regulating the release of these cytokines by T cells.

Similar to SEA-stimulated cells, the levels of IL-4, IL-5, IL-9, and IL-13 release increased 1 year posttreatment in response to SWA stimulation. However, there was no significant difference in these cellular cytokine responses of children who did or did not have infection at 1 year and 2 years posttreatment, again suggesting the absence of T-cell hyporesponsiveness to SWA. In the human host, *S. mansoni* eggs are produced continuously and many are trapped in tissues where they die after a few weeks. In contrast, individual adult worms are estimated to live for 5.7–10.5 years [30]. Therefore, chronically infected humans are continuously exposed to SEA, but only sporadically exposed to otherwise sequestered antigens released from dying worms. This suggests that hyporesponsiveness to SEA, rather than SWA, could be seen as the priority requirement to limit immunopathology.

Treatment causes schistosome worms to disintegrate, exposing the host to these same sequestered adult worm antigens [31], and the other hypothesis for posttreatment increases in type 2 cytokines in response to SWA stimulation is boosting of the recall response via in vivo exposure to antigens [15, 16]. This reliance on exposure to antigen is indicated by multiple treatments, and consequently multiple exposures to antigen, resulting in greater IL-5 and IL-13 production than a single treatment [15]. In the current study, the correlation between the increase in the type 2 cytokine levels to SWA and pretreatment infection intensities suggests that antigen dose upon treatment is crucial to this increase. The follow-up periods of 1 year and 2 years posttreatment are indicative of a long-lived peripheral memory response being key to this increase. The low levels of infection that were treated at 1 year posttreatment may have been insufficient to substantially boost further the memory response, so levels of type 2 cytokines were comparable 1 and 2 years posttreatment.

To conclude, we hypothesize that to limit immunopathology during infection, hyporesponsiveness of the adaptive Th2 response to eggs develops. However, the down-regulation of the adaptive anti-inflammatory response may allow the innate system to respond in a proinflammatory manner. Limiting infection, and hyporesponsiveness, allows the anti-inflammatory Th2 response to develop and at the same time, down-regulation of the proinflammatory response occurs. In contrast, Th2 cell hyporesponsiveness is not as apparent in response to SWA, and increases in type 2 responses posttreatment are likely due to antigen exposure. This difference shows that the human host can down-regulate the potentially tissue-damaging Th2 response to eggs, but with a potential trade-off toward proinflammatory hepatomegaly, while maintaining the ability to mount strong type 2 effector responses when intermittently exposed to adult worm–derived antigens, a process that has been associated with partial immunity to reinfection [13] and can be induced by successive praziquantel treatments [32, 33].

## Supplementary Data

Supplementary materials are available at *The Journal of Infectious Diseases* online (http://jid.oxfordjournals.org/). Supplementary materials consist of data provided by the author that are published to benefit the reader. The posted materials are not copyedited. The contents of all supplementary data are the sole responsibility of the authors. Questions or messages regarding errors should be addressed to the author.

Supplementary Data
