# Supplementary material for: Posttreatment Changes in Cytokines Induced by Schistosoma mansoni Egg and Worm Antigens: Dissociation of Immunity- and Morbidity-Associated Type 2 Responses
Source: J Infect Dis. 2013 Dec 19;209(11):1792–800. doi: 10.1093/infdis/jit826 (PMC4017363; doi:10.1093/infdis/jit826)
Supplement: Supplementary Data [file supp_209_11_1792__index.html]

Posttreatment Changes in Cytokines Induced by Schistosoma mansoni Egg and Worm Antigens: Dissociation of Immunity- and Morbidity-Associated Type 2 Responses — Posttreatment Changes in Cytokines Induced by Schistosoma mansoni Egg and Worm Antigens: Dissociation of Immunity- and Morbidity-Associated Type 2 Responses — Supplementary Data 

# Posttreatment Changes in Cytokines Induced by *Schistosoma mansoni* Egg and Worm Antigens: Dissociation of Immunity- and Morbidity-Associated Type 2 Responses

## Supplementary Data

Supplementary Data

**Files in this Data Supplement:**

- Supplementary Data - Docx file
