# Supplementary material for: Posttreatment Changes in Cytokines Induced by Schistosoma mansoni Egg and Worm Antigens: Dissociation of Immunity- and Morbidity-Associated Type 2 Responses
Source: J Infect Dis. 2013 Dec 19;209(11):1792–800. doi: 10.1093/infdis/jit826 (PMC4017363; doi:10.1093/infdis/jit826)
Supplement: Supplementary Data [file supp_jit826_jit826supp.docx]

|  | Med v SEA | | Med v SWA | | SEA v PHA | | SWA v PHA | | SEA v SWA | |
| --- | --- | --- | --- | --- | --- | --- | --- | --- | --- | --- |
|  | p-value | S-m^a^ | p-value | S-m | p-value | S-m | p-value | S-m | p-value | S-m |
| IL-1β | 2x10^-16^ (<) | 9x10^-4^ | 5x10^-6^ (<) | 0.039 | 6x10^-12^ (<) | 0.027 | 2x10^-16^ (<) | 9x10^-4^ | 2x10^-16^ (>) | 9x10^-4^ |
| IL-12p40 | 9x10^-16^ (<) | 0.021 | 0.022 (>) | 0.045 | 6x10^-12^ (<) | 0.027 | 2x10^-16^ (<) | 9x10^-4^ | 9x10^-16^ (>) | 9x10^-4^ |
| TNFα | 5x10^-16^ (<) | 0.020 | 0.160 | n.s. | 2x10^-15^ (<) | 0.022 | 2x10^-16^ (<) | 9x10^-4^ | 2x10^-11^ (>) | 9x10^-4^ |
| IFNγ | 3x10^-16^ (<) | 0.017 | 2x10^-16^ (<) | 9x10^-4^ | 2x10^-13^ (<) | 0.025 | 2x10^-16^ (<) | 9x10^-4^ | 4x10^-16^ (>) | 0.019 |
| CCL5 | 2x10^-16^ (<) | 9x10^-4^ | 3x10^-5^ (<) | 0.041 | 0.122 | n.s. | 2x10^-16^ (<) | 9x10^-4^ | 3x10^-16^ (>) | 0.017 |
| IL-6 | 2x10^-16^ (<) | 9x10^-4^ | 2x10^-13^ (<) | 0.025 | 0.081 | n.s | 2x10^-16^ (<) | 9x10^-4^ | 2x10^-16^ (>) | 9x10^-4^ |
| IL-10 | 2x10^-16^ (<) | 9x10^-4^ | 2x10^-7^ (<) | 0.037 | 2x10^-11^ (<) | 0.030 | 2x10^-16^ (<) | 9x10^-4^ | 2x10^-16^ (>) | 9x10^-4^ |
| IL-4 | 5x10^-7^ (<) | 0.038 | 2x10^-10^ (<) | 0.032 | 0.111 | n.s. | 0.002 (>) | 0.043 | 2x10^-6^ (<) | 9x10^-4^ |
| IL-5 | 2x10^-13^ (<) | 0.025 | 2x10^-15^ (<) | 0.022 | 0.135 | n.s. | 3x10^-10^ (>) | 0.034 | 4x10^-11^ (<) | 0.031 |
| IL-9 | 0.001 (<) | 0.042 | 2x10^-10^ (<) | 0.032 | 0.045 (<) | 0.045 | 5x10^-6^ (>) | 0.039 | 4x10^-8^ (<) | 0.035 |
| IL-13 | 2x10^-14^ (<) | 0.024 | 2x10^-14^ (<) | 0.024 | 0.003 (<) | 0.044 | 0.003 (>) | 0.046 | 7x10^-8^ (<) | 0.036 |

**Table S1. Statistical significance for comparisons of pre-treatment cytokine release in response to differing stimuli**

^a^S-m: p-value for significance using Simes-modified Bonferroni correction. </> Direction of significance. n.s. = non-significant.

**Table S2. Statistical significance for comparisons of longitudinal cytokine release in response to soluble schistosome antigens**

|  | SEA pre-treatment v 1-yr | | SEA 1-yr v 2-yr | | SWA pre-treatment v 1-yr | | SWA 1-yr v 2-yr | |
| --- | --- | --- | --- | --- | --- | --- | --- | --- |
|  | p-value | S-m | p-value | S-m | p-value | S-m | p-value | S-m |
| IL-1β | 9x10^-13^ (>) | 0.007 | 0.281 | n.s. | - | - | - | - |
| IL-12p40 | 2x10^-4^ (>) | 0.035 | 1x10^-13^ (>) | 0.002 | - | - | - | - |
| TNFα | 3x10^-6^ (>) | 0.028 | 4x10^-8^ (>) | 0.022 | - | - | - | - |
| IFNγ^a^ | 2x10^-6^ (>) | 0.026 | 6x10^-11^ (>) | 0.015 | 0.070 | n.s. | 0.745 | n.s |
| CCL5 | 6x10^-6^ (<) | 0.030 | 9x10^-4^ (>) | 0.039 | - | - | - | - |
| IL-6 | 1x10^-4^ (>) | 0.033 | 2x10^-12^ (>) | 0.011 | 2x10^-14^ (>) | 0.004 | 0.054 | n.s |
| IL-10 | 0.443 | n.s. | 2x10^-9^ (>) | 0.017 | 0.723 | n.s. | 0.048 | 0.025, n.s |
| IL-4 | 9x10^-12^ (<) | 0.013 | 0.002 (<) | 0.043 | 4x10^-13^ (<) | 0.007 | 0.065 | n.s |
| IL-5 | 9x10^-13^ (<) | 0.007 | 0.002 (<) | 0.043 | 1x10^-12^ (<) | 0.011 | 0.070 | n.s |
| IL-9 | 2x10^-10^ (<) | 0.015 | 0.427 (<) | n.s. | 5x10^-9^ (<) | 0.018 | 0.040 | 0.021, n.s. |
| IL-13 | 1x10^-13^ (<) | 0.002 | 3x10^-4^ (<) | 0.037 | 1x10^-12^ (<) | 0.011 | 0.212 | n.s |

^a^SEA-IFNγ pre-treatment v 2yr, p=2x10^-7^, required p = 0.024

**Table S3. Statistical significance for comparisons of type-2 cytokine release in response to soluble schistosome antigens for children with and without *S. mansoni* infection at 1- and 2-yr post-treatment**

|  | SEA 1-yr | | SEA 2-yr | | SWA 1-yr | | SWA 2-yr | |
| --- | --- | --- | --- | --- | --- | --- | --- | --- |
|  | p-value | S-m | p-value | S-m | p-value | S-m | p-value | S-m |
| IL-4 | 0.002 (>)^a^ | 0.019 | 0.100 | n.s | 0.443 | n.s. | 0.935 | n.s. |
| IL-5 | 0.052 | n.s | 4x10^-4^ (>) | 0.013 | 0.214 | n.s. | 0.939 | n.s. |
| IL-9 | 0.011 (>) | 0.031 | 0.007 (>) | 0.025 | 0.125 | n.s. | 0.934 | n.s. |
| IL-13 | 0.013 (>) | 0.037 | 6x10^-6^ (>) | 0.006 | 0.570 | n.s. | 0.472 | n.s. |

^a^*S. mansoni* negative v *S. mansoni* positive
